# Applying Boyer’s Categories: Embracing Digital Creation as a Distinct Form of Scholarship for Promotion and Tenure

**DOI:** 10.1007/s40670-026-02807-3

**Published:** 2026-07-07

**Authors:** Deborah Virant-Young, Seth M. Alexander, Gary L. Beck Dallaghan, Kristina Dzara, Faye Haggar, Kelvin Pollard, Sarah Towner Wright, Gregory M. Polites

**Affiliations:** 1 Clinical Sciences and Biomedical and Translational Sciences Departments, Carle Illinois College of Medicine, Urbana, IL USA; 2 Vanderbilt University School of Medicine, Nashville, TN USA; 3 University of North Carolina School of Medicine, Chapel Hill, NC USA; 4 Carle Illinois College of Medicine, 506 S Mathews Ave, Ste. 180, Urbana, IL 506 S Mathews Ave, Ste. 180 USA; 5 Saint Louis University School of Medicine, Saint Louis, MO USA; 6 Children’s Nebraska, Omaha, NE USA; 7 University of Nebraska Medical Center, Omaha, NE USA; 8 Mercy Primary Care, Saint Louis, MO USA; 9 Health Sciences Library, University of North Carolina at Chapel Hill, Chapel Hill, NC USA; 10 Clinical Sciences, Carle Illinois College of Medicine, Urbana, IL USA; 11 Emergency medicine, WashU Medicine, St. Louis, MO USA

## Abstract

The proliferation of digital platforms has transformed how knowledge is created, shared, and evaluated in academic medicine. Although Boyer’s framework of scholarship has long guided academic recognition, digital scholarship challenges traditional paradigms centered on print publications and conferences. This commentary argues that digital scholarship satisfies Boyer’s framework and should be recognized in promotion and tenure. Digital-first creation, interactivity, and flexibility enable engagement, collaboration, and dissemination, yet institutional recognition remains limited. We propose a framework to evaluate quality, reach, and impact using traditional and alternative metrics, including peer review and engagement analytics, while addressing challenges of literacy, ethics, and intellectual property.

## Introduction

For many faculty members, rising to the rank of full professor marks the pinnacle of a career in academia. Traditionally, joining the ranks of the professoriate is accompanied by expectations of teaching, research, and service. The research component has historically focused on those pursuits classified as the scholarship of discovery [1]. However, in the early 1990 s, Boyer further expanded the concept of scholarly pursuits, labeling them as the scholarship of discovery, integration, application, and teaching. Boyer’s intention was to set forward an evolving definitional framework for scholarship to guide scholars and their institutions alike [1].

As noted, in 1990, Ernest Boyer from the Carnegie Foundation redefined scholarship [1]. For years, the scholarship of discovery seemed to be the gold standard for scholarship. Discovery is building new knowledge through traditional forms of research, such as randomized-control trials. Scholarship of integration interprets the use of knowledge across disciplines. In many ways this was the beginning of Cochrane reviews and formalizing systematic reviews of the literature. Scholarship of application aids society and professions in addressing problems. Scholarship of teaching was new. It presented a new branch of scholarly activity for educators to evaluate their teaching practices to explore what works, what does not, and how to improve.

Over the years, the academy has embraced these four categories of scholarship to stratify the work of faculty and trainees. Others have expanded on this work by refining what scholarship looks like as well as how to appraise it [2, 3]. In recent years, the proliferation and dissemination of digital media in all its forms have created new modalities with which to discover, integrate, apply, and teach. This “digital scholarship” is unique in that it is conceived, created, and disseminated in a virtual environment. As compared with traditional forms of scholarship, digital scholarship offers unique opportunities for rapid, global engagement. The challenge lies in identifying educational leaders who recognize the merits of digital scholarship. Johng and colleagues sought to explore how institutions recognized digital scholarship by reviewing publicly available promotion and tenure guidelines, finding that only 12 of 148 institutions explicitly endorsed digital scholarship for purposes of promotion [4].

These findings are not surprising. The hallmark of scholarly activity continues to be publication in peer-reviewed journals. Digital artifacts, the creations of digital scholarship such as moderated discussion forums or software, bypass traditional forms of dissemination (e.g., journal articles, conference presentations) [2]. The swiftness with which digital work can be fast tracked online leads some to believe that digital artifacts consistently lack “rigorous” standards established for traditional forms of scholarship, such as adequate preparation, peer review, and reflection [3]. However, numerous digital initiatives have been rigorously evaluated and have been published in more traditional forums [5]. In 2015, consensus criteria for social media scholarship were published, which emphasized that transparent, critical appraisal is essential [6]. Additionally, Maldonado and colleagues report various methods of evaluation for the quality of digital scholarship, such as the social media index or the Medical Education Translational Resources: Impact and Quality score [7–9].

Even though the credibility of digital scholarship has been explored in the context of Boyer’s four categories, its role in the evaluation of faculty promotion remains ill-defined [6, 10]. We argue that, due to the dynamic nature of digital artifacts, scholarship in the digital space can satisfy Boyer’s criteria and should be recognized as such. This commentary argues for the legitimization of this form of scholarship and proposes ideas for how academic leaders can determine the quality, reach, and impact of digital artifacts so as to recognize faculty efforts in this space.

## Defining Characteristics of Digital Scholarship

The literature proposes multiple approaches for digital scholarship. These include the creation of digital artifacts and the development of online platforms for scholarly discussion and information sharing. Digital innovations may range from interactive, three-dimensional, anatomical models to adaptive learning algorithms tailored for diverse clinical educational settings. The key is recognizing the scholarship and originality within the creation of these artifacts rather than simply the work accomplished through their application and dissemination. With such a range of artifacts, it is critical to define such works and provide a clear structure for how they are presented and appraised alongside other scholarly work within an educator’s portfolio [11, 12].

### Digital-First Creation

Digital scholarship employs a “digital-first” approach in which scholarly content is intentionally created, engaged with, or disseminated through online formats [11]. This approach encourages the development of scholarly products specifically for use within digitally informed academic communities. Examples include digital artifacts such as “digital electives” that teach technical skills—illustrating Boyer’s Scholarship of Teaching [1]. Fu and Zuckerman describe an undergraduate medical school elective, Digital MD, which trains students in social media and digital engagement in medicine [12], reflecting Boyer’s Scholarship of Application [1]. Although such work currently appears in traditional journals, we argue that the processes and structures used to create these projects along with evaluation and reflection embody a distinctly “digital-first” model.

Similarly, Shah and colleagues highlight the transformative role of digital platforms to foster knowledge dissemination and professional engagement in medical education [13]. A defining feature of a “digital-first” approach is its emphasis on interactivity and engagement. Unlike traditional scholarly outputs, digital formats offer real-time discourse, collaborative learning, and broader audience reach. Breu and Cooper advocated for the inclusion of short, educational tutorials shared on social media, referred to as “tweetorials,” in academic promotion portfolios [14]. The “tweetorials” represent the scholarship of teaching achieved through social media, where reach and impact of dissemination may be detailed and articulated. Similarly, El Hussein and colleagues illustrate how scholars leverage digital platforms for innovative educational strategies and professional networking through the creation of interactive digital communities [15].

### Fluidity and Flexibility

Digital scholarship requires fluidity and flexibility, enabling scholars and their audience to continuously engage with scholarly work in new ways. Use of comment fields on blogs or live chats on X.com exemplify this dynamic process. Chan and colleagues discussed systems that support scholarly work involving social media, underscoring that adaptability and systematic growth is required for effective digital engagement [16]. Acquaviva and colleagues, in a concurring piece, emphasized the significance of adapting guidelines to include documenting professional social media engagement as a form of scholarship, reflecting the evolving landscape of academic recognition in the digital era [17].

### Ease of Access

Access to content is fundamental to digital scholarship, promoting inclusivity and accessibility. The integration of open access digital scholarship within dental education emphasizes the importance of open resources in academic training to reduce inequities in access [18]. Goh and Sandars further argue for rethinking educational scholarship in the digital world, advocating for widespread access to the knowledge it creates and shares [19]. This extends beyond the open-access publications of traditional journals to include ease and equality of access through digitally informed scholarly communities.

Although digital scholarship offers ease of access compared with traditional print or subscription-based dissemination, this advantage is not uniform across all contexts. Pockets of limited internet access remain globally, shaped by geography, infrastructure, and affordability. Rural and underserved communities in the United States continue to experience significant broadband gaps, with reduced capacity for reliable, high-speed connectivity that can limit engagement with online scholarly content [20, 21]. Globally, nearly three billion people still lack consistent internet access, and even where connectivity exists, cost, gender inequities, and platform restrictions can constrain meaningful participation in digital knowledge ecosystems [22]. Importantly, these inequities are not unique to digital scholarship; they mirror longstanding disparities in access to academic journals, conferences, and libraries that have historically privileged well-resourced institutions [23, 24]. Recognizing digital scholarship within promotion and tenure should therefore be accompanied by intentional strategies, such as low-bandwidth formats, open-access dissemination, and institutional support for infrastructure development, to ensure that expanded recognition of digital artifacts advances, rather than exacerbates, equity in scholarly communication.

## Evaluating Digital Scholarship

As digital scholarship evolves, traditional paradigms of academic output are challenged. Digital scholarship has the potential to reshape how knowledge is produced and shared but also necessitates rethinking how such work is evaluated. The adaptive, participatory, and decentralized nature of digital scholarship calls for creative frameworks that uphold standards for quality scholarship. In the following section, we explore methods for evaluating digital scholarship, with a focus on ensuring rigor, relevance, and recognition within professional contexts.

### Metrics of Impact

Digital scholarship must be rigorously evaluated to determine its impact. The acknowledgement of digital scholarship itself is wholly insufficient to meet Boyer’s criteria for scholarship [1]. It must also meet expectations of originality, building upon theory, being archived and disseminated, and allowing for comment and feedback [6, 25, 26]. Some standards, such as those requiring originality and that the work build upon theory, should be upheld to ensure that work is high-quality and evidence-supported [26]. In the digital era, other standards such as archiving work, disseminating digital artifacts, and offering opportunities for public comment will need to be considered in drastically broader contexts. Transparent, flexible, field- and institution- specific guidelines for those engaging in digital scholarship will be essential for their evaluation.

In a digitally informed scholarly community, data on the reach and impact of digital content will be useful in its appraisal [11, 19]. The exact mechanism for this analysis is a source of debate. Altmetric is one relatively well known metric produced by a private company as a way to evaluate digital scholarship based on engagement and digital distribution [27]. Alternative article-level metrics such as citation count have also been suggested [28]. Social media also offers new metrics, such as page views, that can represent the dissemination and impact of digital scholarship [6, 27]. These metrics can also include more diverse aspects of social media engagements (likes, comments) or impressions [29].

Evaluating digital scholarship requires a comprehensive approach that combines traditional and alternative metrics to capture its value and impact [29, 30]. No single measure is sufficient; faculty should therefore use multiple metrics to demonstrate the significance of their work. For example, social network analysis can reveal the reach and engagement of digital content by identifying key interactions and geographic distribution during a live social media chat [30]. While these metrics help describe impact, assessing the quality and scope of digital scholarship often falls to the reader, who must critically appraise the work by considering factors such as the author’s expertise and the originality of the contribution [20]. High-quality digital scholarship should clearly state its purpose, build on existing literature and theory, and show how it extends or enhances prior work to reduce the burden of appraisal on the reader.

### Evaluation Frameworks

Colmers and colleagues established comprehensive quality checklists for health professions education blogs and podcasts [30]. Their approach identified over 150 potential quality indicators, subsequently refined to develop core indicators across three essential domains: credibility establishment, content quality, and design effectiveness. Similarly, Lin and colleagues developed a framework specifically targeting blogs and podcasts in health professions education, organizing quality indicators into comparable domains that emphasize credibility verification, content assessment, and design evaluation [31]. These tools represent progress toward standardizing digital scholarship assessment, yet their implementation remains inconsistent across academic disciplines and among academic institutions. The frameworks do share a common emphasis, however, suggesting a convergence around essential quality markers despite the diversity of digital formats.

The impact of digital scholarship must be closely tied to rigorous means of evaluation. Other structured approaches to evaluating digital scholarship have been described. Chan and colleagues developed and offered validity evidence for the ALiEM AIR (Academic Life in Emergency Medicine Approved Instructional Resources) for assessing Free Open Access Medical Education (FOAMed) resources [32]. They demonstrated that the tool achieves moderate to high reliability when implemented with multiple raters. The ALiEM AIR framework evaluates dimensions including accuracy of information, evidence-based content organization, and educational utility.

Table 1 offers a comparison of these frameworks. As can be seen, no one framework may be complete. Several of them identify quality markers [30–32]. For reach and impact, SMI [8] indicates measures to use, but bases results on popularity metrics. Because of this, use of multiple forms of evaluation may be appropriate as a measure of quality and impact of digital scholarship.

**Table 1:** Frameworks to evaluate quality of digital creations

Feature	ALIEM AIR [9, 32]	Colmers et al. [30]		Lin et al. [31]	SMI [8]		METRIQ Score [32]
Purpose	FOAM content evaluation (Emergency Medicine)	Quality appraisal via checklists		Identify quality indicators for digital content	Quantify reach and visibility		Evaluate quality and impact of online resources
Framework Type	Scoring system with known validity evidence	Consensus-based checklists		Modified Delphi indicator list	Popularity metric		Structured, scoring tool with validity evidence
Key Components	Accuracy, EBM, BEEM score, references	Credibility, content, design		Transparency, accuracy, design	Web traffic, social metrics		Transparency, referencing, author identity, utility
Validity Evidence	IRR and Kane’s framework; moderately correlated with expert gestalt	Delphi consensus among creators and educators		> 90% consensus among educators	No formal validity evidence collected		Psychometric validation through expert rating
Strengths	High reliability for targeted FOAM evaluation	Simple and applicable across platforms		Flexible and easily integrated	Reflects influence and presence		Combines rigor and practical use
Weaknesses	Requires multiple trained raters		No formal scoring rubric	No formal validity evidence collected	Not a measure of quality	Does not measure reach or visibility	
Best Use Case	Resource vetting for EM residency programs		Editorial boards, creators, peer reviewers	Curriculum development and review	FOAM brand tracking, visibility	FOAM resource quality review and guidance	

*ALIEM AIR* Academic Life in Emergency Medicine Approved Instructional Resources, *SMI* Social Media Index, *EBM* Evidence-based medicine, *BEEM* Best Evidence in Emergency Medicine, *FOAM* Free Open Access Medical Education, *IRR* Inter-rater Reliability

### Peer Review

As frameworks to evaluate digital scholarship evolve, evidence of critical appraisal will be a necessary part of the scholarly evolution [6]. This appraisal should, as many traditional manuscripts do, include a reflective critique conducted by the author(s) themselves, to support iterative quality improvement, especially for longitudinal scholarship. Moreover, peer review, the standard for traditional scholarship, is highly recommended for digital scholarship [25, 26, 31].

Establishing a peer review process can offer a level of rigor expected of traditional forms of academic scholarship. One example of digital scholarship with an established peer-review process is the Harvard Macy Blog, which has an editor-in-chief and one community member review each post [33]. The Saint Louis University School of Medicine Center for Educator Development, Advancement, and Research (CEDAR) Community Blog has a nine member peer-review board of faculty, staff and trainees, such that each post is reviewed by the editor-in-chief and two board members prior to publication on the web [34]. More recently, #MedEdChat transitioned to a blog format with a peer-review component. The Alliance for Clinical Education briefs serve as another example of digital scholarship with peer review [35]. Because these examples are peer reviewed, authors can denote them on their curriculum vitaes with substantially higher credibility than solely self-created and disseminated materials.

### Sustainability and Longevity

Rapid feedback and wide dissemination are key advantages of digital scholarship [7]. Public, accessible dissemination is important to meet criteria for digital scholarship [6, 26], allowing it to reach a broader audience than more traditional forums [29]. Social media, as one diverse example, can increase the visibility of digital scholarship by allowing for more active engagement and discussion than simply publishing a traditional paper online [4, 36]. Ideally, digital scholarship should be archived in a publicly accessible long-term location. One method of doing this would be to utilize permalinks (permanent links), which are unique and unchanging URLs for the website where the digital artifact is housed [37].

In considering the dissemination, appraisal, and revision of scholarship in the digital age, online platforms and communities must provide centralized support for scholarly endeavors. As described above, digitally informed scholarly communities serve as a key means for distribution and collaboration for digital scholars and their work. As an early adopter of this strategy, social media has allowed for the free, speedy, and open distribution of ideas between scholars and fostered a sense of community [38, 39]. This can be especially true during challenging circumstances such as the rapid transition to virtual learning during the early COVID-19 pandemic, where #HMIchat repurposed their existing hashtag to crowdsource content related to timely teaching and learning concerns [40]. The creation and moderation of these communities is a form of digital scholarship in itself (Scholarship of Integration [1]). Online communities and authors will have to play an active and instrumental role in protecting the creators and beneficiaries of digital scholarship by refereeing intricate questions related to data transparency, misinformation, preventing plagiarism, and privacy [41].

## Challenges to Consider

Up to this point, we have supported the application of Boyer’s categories of scholarship to include digital scholarship [1]. However, the integration of digital forms of scholarship introduces significant challenges that extend beyond institutional recognition to encompass digital literacy, ethical use of emerging technologies such as artificial intelligence (AI), and the safeguarding of intellectual property.

Institutional recognition remains the most visible hurdle, manifesting in review, promotion, and tenure processes [28]. Current structures privilege research and publications in peer-reviewed journals [41, 42]. Although tracks for educators and educational scholarship exist [43], these largely hinge on traditional forms of scholarship, and emphasis on scholarship can conflict with the work academicians predominantly undertake: teaching, patient care, and service. While some institutions have begun accepting metrics aligned with digital scholarship, these metrics are new and may be unfamiliar. Given that the scholarship of teaching is itself a relatively recent concept [1, 2] and continues to face barriers to full acceptance, the recognition of digital scholarship will require sustained cultural and structural change.

Beyond recognition, digital literacy is foundational. Without deliberate efforts to equip faculty and learners with the skills to critically evaluate, create, and disseminate digital artifacts, an expanded definition of scholarship risks amplifying inequities. Those in digitally fluent communities or younger generations may disproportionately benefit [44]. Moreover, the rapid adoption of AI-driven tools for content creation and analysis introduces ethical complexities: transparency in authorship, avoidance of algorithmic bias, and accountability for outputs generated by non-human agents must be addressed. Institutions should embed digital literacy and AI ethics into faculty development and curricular frameworks to ensure responsible engagement with these technologies.

The ethical dimension of digital scholarship extends beyond technical fluency. Scholars must navigate questions of accuracy, validity, and harm prevention in an environment where misinformation can spread rapidly. Mechanisms for correcting errors—whether human or AI-generated—are essential to maintain public trust in scholarly work. Additionally, as digital platforms blur boundaries between formal research and public discourse, guidelines for privacy, consent, and data security must evolve alongside technology [42]. Institutional Review Boards should adapt criteria to avoid stifling scholarship while safeguarding participants.

Finally, intellectual property rights demand renewed attention. Digital dissemination and AI-assisted creation challenge traditional notions of ownership. Copyright, patents, and trademarks are increasingly vulnerable in an era of automated content generation and global sharing. Legal disputes over infringement and digital piracy underscore the need for proactive strategies: faculty development on IP law, technological safeguards, and institutional access to legal expertise [43]. Furthermore, as generative AI tools become commonplace, questions of authorship and originality will require clear policies to prevent ethical and legal ambiguities.

In sum, the evolution of scholarship into digital domains is not merely a technical shift—it is a cultural, ethical, and legal transformation. Institutions must invest in frameworks that promote digital literacy, uphold ethical standards in AI use, and protect intellectual property. This then will we ensure that digital scholarship strengthens, rather than undermines, the integrity of academic work.

### Embracing Digital Scholarship

In considering the future of scholarly activities, the recognition of digital scholarship not only legitimizes current work but it presents opportunities for faculty development, scholarly collaborations, and rapid dissemination [6, 42]. For academia to remain relevant in an increasingly digital and interconnected world, the inclusion of the digital scholarship symbolizes an evolution for the modern age and beyond. Academic medicine should champion this movement so that institutions legitimize it as a meaningful academic pursuit [5].

Digital scholarship facilitates opportunities for collaboration, not only locally but globally [36, 38, 43]. By embracing digital scholarship as a valid form of academic work, institutions empower faculty to forge connections and collaborate in unprecedented ways. Where international collaborations were once impractical aside from email-based collaborations, digital platforms bridge the distance, allowing scholars to expand the scope of their work. As Luc and colleagues note, dynamic social media activities engage individuals from different backgrounds, generating diverse perspectives and innovative approaches to problem-solving [42]. Imagine networks uniquely suited to tackling complex problems that traditional methods may eventually complete, but that take so much longer due to physical constraints. Such partnerships could equitably engage historically marginalized corners of the globe in the scholarly debate.

The irony of publishing a traditional, peer-reviewed article advocating for digital scholarship is not lost on the authors. In this new age, faculty have the ability to share with a global audience instantly, eliminating lengthy delays of traditional publishing cycles. It is important to share rapidly so as to contribute to relevant scholarly conversations, particularly when critical, timely topics need to be addressed. The recent experiences stemming from the COVID-19 pandemic offer a good example of the need for sharing in a timelier manner and offer a cautionary tale about the risks of misinformation. Digital platforms enable greater audience engagement as well, creating dynamic, interactive communities of practice.

Institutions need to formalize inclusion of the digital scholarship, rewarding faculty for their efforts in this domain. Inclusion in promotion and tenure guidelines signals it is a legitimate, respected form of scholarship [6, 16]. Based on the metrics we have discussed above, Table 2 suggests a breakdown of promotion guidelines by rank institutions can use as a starting point.

**Table 2:** Criteria for promotion and tenure

Rank	**Assistant Professor** ^**1**^
Creation Competence	Development of digital artifacts that apply sound scholarly criteria
Emerging Audience	Evidence of growing engagement metrics (i.e., 100–500 views, downloads)
Early Validation	Quality peer feedback through comments, citations, or adoption of materials by others
Professional Development	Participation in digital scholarship training and evidence of engagement in communities of practice
Quality Standards	Examples of adherence to guidelines for digital creation in their modality of choice
Rank	**Associate Professor** ^**2**^
Sustained Production	Demonstrates regular creation of digital artifacts that build upon one another in a coherent scholarly narrative
Significant Reach	Substantial engagement metrics (1,000 + views/downloads) or evidence of adoption by other institutions
Peer Review	Formal peer review of digital creations
Recognition	Invited to present on digital scholarship expertise (locally or regionally)
Field Influence	Evidence that work has influenced practice and thinking in the field
Mentorship	Evidence of guiding colleagues in digital scholarship practices
Rank	**Professor** ^**3**^
Transformative Impact	Evidence that digital scholarship has influenced practices, policies, procedures
Exceptional Reach	Major engagement metrics (2,500 + views/downloads) or widespread adoption across multiple institutions
Leadership	Development of platforms, standards, or frameworks that enable others’ digital scholarship
Recognition	Recognized through awards for work, invited presentations (national or international), deemed authoritative expert of digital creation
Community Building	Creation or leadership of digital scholarly communities

^1^Committees should recognize early-career digital scholars are building foundations and establishing their digital footprint

^2^Committees should evaluate the developing reputation of a digital scholar whose work shows depth, coherence, and meaningful impact

^3^Committees should evaluate national and/or international endorsement as evidence of digital scholarship recognized for excellence, innovation and significant impact.

## Conclusion

The characteristics of digital scholarship including digital-first creation, interactivity and engagement, fluidity and flexibility, and open access, demonstrate its transformative potential for academic medicine. By embracing these principles, scholars can enhance the accessibility, impact, and evolution of knowledge dissemination in the digital age.

Digital scholarship represents a fundamental evolution in how we conceptualize academic work. By embracing digital scholarship, institutions will acknowledge the unique opportunities and challenges of digital creation for faculty. The challenge of institutional recognition must be resolved to move forward. As time goes on, faculty who engage in digital scholarship will pioneer new forms of knowledge creation that are more accessible, collaborative, and responsive than ever before. Promotion and tenure committees that develop thoughtful, rank-appropriate standards for evaluating this work have the potential to attract the best and brightest faculty to this space.

## Declarations

### AI Usage

We used Microsoft Copilot to assist with editing portions of the manuscript to improve the language. Final edits have been done by the writing team.
